# Improving deep learning method for biomedical named entity recognition by using entity definition information

**DOI:** 10.1186/s12859-021-04236-y

**Published:** 2021-12-17

**Authors:** Ying Xiong, Shuai Chen, Buzhou Tang, Qingcai Chen, Xiaolong Wang, Jun Yan, Yi Zhou

**Affiliations:** 1 Department of Computer Science, Harbin Institute of Technology, Shenzhen, Shenzhen, 518055 China; 2 Peng Cheng Laboratory, Shenzhen, China; 3 Yidu Cloud (Beijing) Technology Co., Ltd, Beijing, China; 4 Zhongshan School of Medicine, Sun Yat-Sen University, Guangzhou, 510080 China

**Keywords:** Biomedical named entity recognition, Entity definition information, Machine reading comprehension, Span-level one-pass method

## Abstract

**Background:**

Biomedical named entity recognition (NER) is a fundamental task of biomedical text mining that finds the boundaries of entity mentions in biomedical text and determines their entity type. To accelerate the development of biomedical NER techniques in Spanish, the PharmaCoNER organizers launched a competition to recognize pharmacological substances, compounds, and proteins. Biomedical NER is usually recognized as a sequence labeling task, and almost all state-of-the-art sequence labeling methods ignore the meaning of different entity types. In this paper, we investigate some methods to introduce the meaning of entity types in deep learning methods for biomedical NER and apply them to the PharmaCoNER 2019 challenge. The meaning of each entity type is represented by its definition information.

**Material and method:**

We investigate how to use entity definition information in the following two methods: (1) SQuad-style machine reading comprehension (MRC) methods that treat entity definition information as query and biomedical text as context and predict answer spans as entities. (2) Span-level one-pass (SOne) methods that predict entity spans of one type by one type and introduce entity type meaning, which is represented by entity definition information. All models are trained and tested on the PharmaCoNER 2019 corpus, and their performance is evaluated by strict micro-average precision, recall, and F1-score.

**Results:**

Entity definition information brings improvements to both SQuad-style MRC and SOne methods by about 0.003 in micro-averaged F1-score. The SQuad-style MRC model using entity definition information as query achieves the best performance with a micro-averaged precision of 0.9225, a recall of 0.9050, and an F1-score of 0.9137, respectively. It outperforms the best model of the PharmaCoNER 2019 challenge by 0.0032 in F1-score. Compared with the state-of-the-art model without using manually-crafted features, our model obtains a 1% improvement in F1-score, which is significant. These results indicate that entity definition information is useful for deep learning methods on biomedical NER.

**Conclusion:**

Our entity definition information enhanced models achieve the state-of-the-art micro-average F1 score of 0.9137, which implies that entity definition information has a positive impact on biomedical NER detection. In the future, we will explore more entity definition information from knowledge graph.

## Background

**Fig. 1:**
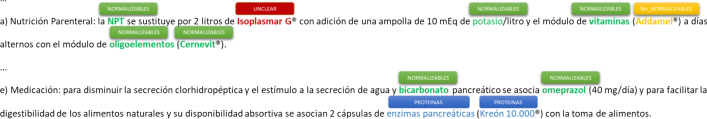
Examples of the biomedical named entities in Spanish records. (NORMALIZABLE entities in green, PROTEINAS entities in blue, NO_NORMALIZALLE entities in yellow and UNCLEAR entities in red. Notice that UNCLEAR entities are not included in the final evaluation.)

Biomedical named entity recognition (NER) is a fundamental task of biomedical text mining to identify biomedical entity mentions of different types in biomedical text. Most biomedical NER studies focus on the biomedical text in English. To accelerate the development of Spanish biomedical NER techniques, Martin Krallinger et al. organized a specific challenge for chemical & drug mention recognition in Spanish biomedical text, called PharmaCoNER, in 2019 [1]. Participants were required to recognize the entities in Spanish biomedical text, as shown in Fig. 1.

Biomedical NER is a typical sequence labeling problem, and lots of state-of-the-art methods have been proposed for this problem, such as BiLSTM-CRF [2]. Almost all these methods do not consider the meaning of different entity types, which may benefit biomedical NER. The meaning of each entity type can be represented by its definition. For example, the definition of PROTEINAS in the guideline of PharmaCoNER 2019 is: **“**Las menciones de proteínas y genes incluyen péptidos, hormonas peptídicas y anticuerpos.” (Protein and gene mentions include peptides, peptide hormones, and antibodies). In this paper, we explore how to encode entity definition information in two kinds of deep learning methods for NER. They are: (1) SQuad-style MRC methods designed to find a continuous span of entity mentions in given text for each type. We use each type's entity definition as a query instead of a naive query generated by simple rules in MRC methods. For convenience, we adopt MRC to represent SQuad-style MRC in the following sections in this paper. (2) Span-level one-pass (SOne) methods that predict entity spans of one type by one type. We use entity definition information to represent each entity type's meaning and introduce the entity type meaning into SOne. The definition information of each type includes the original definition of each type in the guideline and entity mentions in the text. We compare them in the SOne model.

In order to evaluate the performances of MRC and SOne, we conduct experiments on the PharmaCoNER 2019 corpus. Experiments show that the entity definition information brings improvements to both MRC and SOne methods. The improvement in micro-averaged F1-score is about 0.003. The MRC method using entity definition information as query achieves the best performance with a micro-average precision of 0.9225, a recall of 0.9050, and an F1-score of 0.9137, respectively. It outperforms the best model of the PharmaCoNER 2019 challenge by 0.0032 in micro-averaged F1-score.

## Related work

The natural language processing (NLP) community has made a great contribution to the development of NER in the biomedical text through challenges, such as I2B2 (Informatics for Integrating Biology and the Bedside) [3, 4], BioCreative (Critical Assessment of Information Extraction systems in Biology) [5, 6], SemEval (Semantic Evaluation) [7, 8], CCKS (China Conference on Knowledge Graph and Semantic Computing) [9, 10] and IberLEF [11]. A large number of methods have been proposed for biomedical NER. Most of them can be classified into the following three categories: (1) Rule-based methods that extract named entities using specific rules design by experts. The earlier clinical NLP tools are rule-based systems relying on clinical dictionaries, such as MedLEE [12], KnowledgeMap [13] and MetaMap [14]. (2) Supervised machine learning methods with hand-crafted features Maximum Entropy (ME) [15, 16], Support Vector Machines (SVM) [17], CRF [18, 19], Hidden Markov Models (HMM) [20, 21] and Structural Support Vector Machines (SSVM) [22]. They usually treat NER as a sequence labeling task, which tags a sentence with a label sequence. The common features used in the supervised machine learning methods include orthographic information (e.g. capitalization, prefix, suffix and word-shape), syntactic information (e.g., POS tags), dictionary information, n-gram information, disclosure information (e.g. section information in EHRs) and some features generated from unsupervised learning methods [23]. (3) Deep learning methods that can learn features from large unlabeled data without costly feature engineering. Convolutional Neural network (CNN) [24], Recurrent Neural Network (RNN) [25] and Long Short Term Memory neural network (LSTM) [2] have been widely used for biomedical NER and show good performance. Besides the methods mentioned above, there are also some other attempts. For example, to tackle the low-resource problem in the biomedical domain, researchers introduce multi-task learning methods to learn more abundant information from other tasks, such as NER from other sources, chunking, and POS tagging [26–28], and deploy transfer learning methods to first learn knowledge from related sources and then finetune on target [29–33].

Nowadays, there is an upward trend in defining NLP tasks in the MRC framework. MRC models [34–36] extract answer spans from the context given a pre-defined question. Generally, SQuad-style MRC models can be formalized as predicting the start position and the end position of the answer. Li et al. [37] treat the entity-relation extraction task as a multi-turn question answering and propose a unified MRC framework to recognize entities and extract relationships. Li et al. [38] propose an MRC method to recognize both flat and nested entities.

## Material and methods

### Datasets

In this study, all experiments are conducted on the PharmaCoNER 2019 corpus annotated by medicinal chemistry experts according to a pre-defined guideline. The corpus contains 1000 clinical records with 24,654 chemical & drug mentions. The corpus is divided into a training set of 500 records, a development set of 250 records and a test set of 250 records, where the test set is hidden in a background set of 3751 records during the test stage of the competition. In experiments, we first split each record into sentences by sentence ending symbols, including ‘\n’, ‘.’, ‘;’, ‘?’, and ‘!’. About 95% of sentences are no longer than 230 tokens. The corpus statistics, including the number of records, sentences, and chemical & drug mentions of different types, are listed in Table 1. It should be noted that the UNCLEAR mentions are not considered during the competition.

**Table 1:** Statistics of the PharmaCoNER 2019 Corpus

Statistic	#Training	#Development	#Test	#Background
RECORDS	500	250	250	3751
SENTENCES	8776	4028	4260	\
NORMALIZABLES	2304	1121	973	\
NO_NORMALIZABLES	24	16	10	\
PROTEINAS	1405	745	859	\
UNCLEAR	89	44	0	\

### Task definition

Given a sequence $X = \left\{ {x_{1} ,x_{2} , \ldots ,x_{n} } \right\}$ of length *n*, we need to assign a label sequence $Y = \left\{ {y_{1} ,y_{2} , \ldots , y_{n} } \right\}$ to *X*, where *y*_*i*_ is the possible label of token *x*_*i*_ (1 $\le i \le n$) (e.g., PROTEINAS, NORMALIZABLES, NO_NORMALIZABLES, UNCLEAR).

**MRC definition:** the sequence labeling problem can be redefined in the MRC framework as follows, For each label type *y*, its definition information is regarded as a query $q^{y} = \left\{ {q_{0} ,q_{1} , \ldots ,q_{m} } \right\}$ of length *m*, a sentence *X* is regarded as the context of $q^{y}$, the span of an entity of type *y*, and $x_{start:end}^{y} = \left\{ {x_{start} ,x_{start + 1} , \ldots ,x_{end - 1} , x_{end} } \right\}$, is recognized as an answer. Then, the original sequence labeling problem can be represented by ${ }\left( {q^{y} ,X, x_{start:end}^{y} } \right)$. The goal of MRC is to find the spans of all entity mentions of all types, given all sentences.

**SOne definition:** SOne takes sequence *X* as inputs and predicts the spans of all entities of one type by one type using a multi-layer pointer network [39]. The number of network layers depends on the number of entity types. For each type of entity, we add entity definition information *e* to enhance SOne by concatenating it to all tokens.

### Query generation for MRC

Query generation is critical for MRC, since queries usually contain some prior knowledge (e.g. entity type definition) about tasks. Li et al. [40] introduce various kinds of query generation methods, including keywords, Wikipedia, rule-based template filling, synonyms, keywords combined synonyms and annotation guideline notes, and compare them. The results show that annotation guideline is the best choice for query generation. Following Li et al. [40], we compare two kinds of query generation: annotation guideline and rule-based template filling. Table 2 shows our generated queries for each type of entity.

**Table 2:** Generated queries for each type of entity

Entity type	Query type	Generated query
PROTEINAS	Guideline	**“Las menciones de proteínas y genes incluyen péptidos, hormonas peptídicas y anticuerpos.”** (Protein and gene mentions include peptides, peptide hormones, and antibodies.)
	Rule-template	**¿Qué entidades PROTEINAS se mencionan en el texto?** (Which PROTEINAS entities are mentioned in the text?)
NORMALIZABLES	Guideline	**“Menciones de productos químicos que pueden normalizarse manualmente a un identificador de concepto único.”** (Chemical mentions that can be manually normalized to a unique concept identifier.)
	Rule-template	**¿Qué entidades NORMALIZABLES se mencionan en el texto?** (Which NORMALIZABLE entities are mentioned in the text?)
NO_NORMALIZABLES	Guideline	**“Menciones de productos químicos que no se pudieron normalizar manualmente a un identificador de concepto único.”** (Chemical mentions that could not be manually normalized to a unique concept identifier.)
	Rule-template	**¿Qué entidades No_NORMALIZABLES se mencionan en el texto?** (Which NON-NORMALIZABLE entities are mentioned in the text?)
UNCLEAR	Guideline	**“Casos de menciones generales de clase de sustancias de relevancia clínica y biomédica, incluidas ciertas formulaciones farmacéuticas, tratamientos generales, programas de quimioterapia, vacunas y un conjunto predefinido de sustancias generales ( por ejemplo: Estragón, Silimarina, Bromelaína, Melanina, Vaselina, Lanolina, Alcohol, Tabaco, Marihuana, cannabis, opio y gluten).”** (Cases of general mentions of class of substances of clinical and biomedical relevance, including certain pharmaceutical formulations, general treatments, chemotherapy programs, vaccines and a predefined set of general substances (for example: Tarragon, Silymarin, Bromelain, Melanin, Vaseline, Lanolin, Alcohol, Tobacco, Marijuana, cannabis, opium and gluten))
	Rule-template	**¿Qué entidades UNCLEAR se mencionan en el texto?** (Which UNCLEAR entities are mentioned in the text?)

### Model detail

**Fig. 2:**
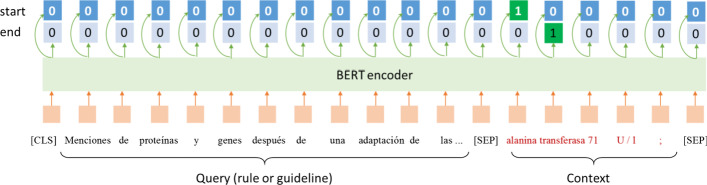
SQuad-style MRC (denoted as MRC) model for NER

In this study, We utilize BERT (Bidirectional Encoder Representations from Transformers) [41] as our model backbone. Figure 2 shows the skeleton of the MRC model. Given query $q^{y}$ and sentence *X*, we need to predict the span of every entity of type *y*, including a start position $x_{start}^{y}$ and an end position $x_{end}^{y}$. The model first takes the following input and encodes it by BERT:

1 $$input_{MRC} = \left\{ {\left[ {CLS} \right], q^{y} ,\left[ {SEP} \right], X,\left[ {SEP} \right]} \right\},$$

where $\left[ {CLS} \right]$ and $\left[ {SEP} \right]$ are special tokens of BERT, denoting whole sentence and sentence separator, respectively. Suppose that the last layer output of BERT is ${\rm H} \in {\mathbb{R}}^{s \times d}$, where *s* is the total length of $\left[ {CLS} \right]$, $q^{y}$, $\left[ {SEP} \right]$, *X* and $\left[ {SEP} \right]$, and *d* is the dimension of the last layer output of BERT, the model then predicts the possibilities of start position and end position as follows:

2 $$P_{start} = softmax\left( {{\rm H} \cdot W_{start} + b_{start} } \right) \in {\mathbb{R}}^{m \times 2} ,$$

3 $$P_{end} = softmax\left( {{\rm H} \cdot W_{end} + b_{end} } \right) \in {\mathbb{R}}^{m \times 2} ,$$

where $W_{start}$ and $W_{end}$ are trainable parameters, $b_{start}$ and $b_{end}$ are biases.

The predicted start index $I_{start}$ and end index $I_{end}$ are:

4 $$I_{start} = \left\{ {j{|}argmax\left( {P_{start}^{j} } \right) = 1, j = 1,2,3, \ldots ,m} \right\}$$

5 $$I_{end} = \left\{ {k{|}argmax\left( {P_{end}^{k} } \right) = 1, k = 1,2,3, \ldots ,m} \right\}$$

We use MRC_rule and MRC_guideline to denote MRC using rule-based template filling for query generation and MRC using annotation guideline as query, respectively.

**Fig. 3:**
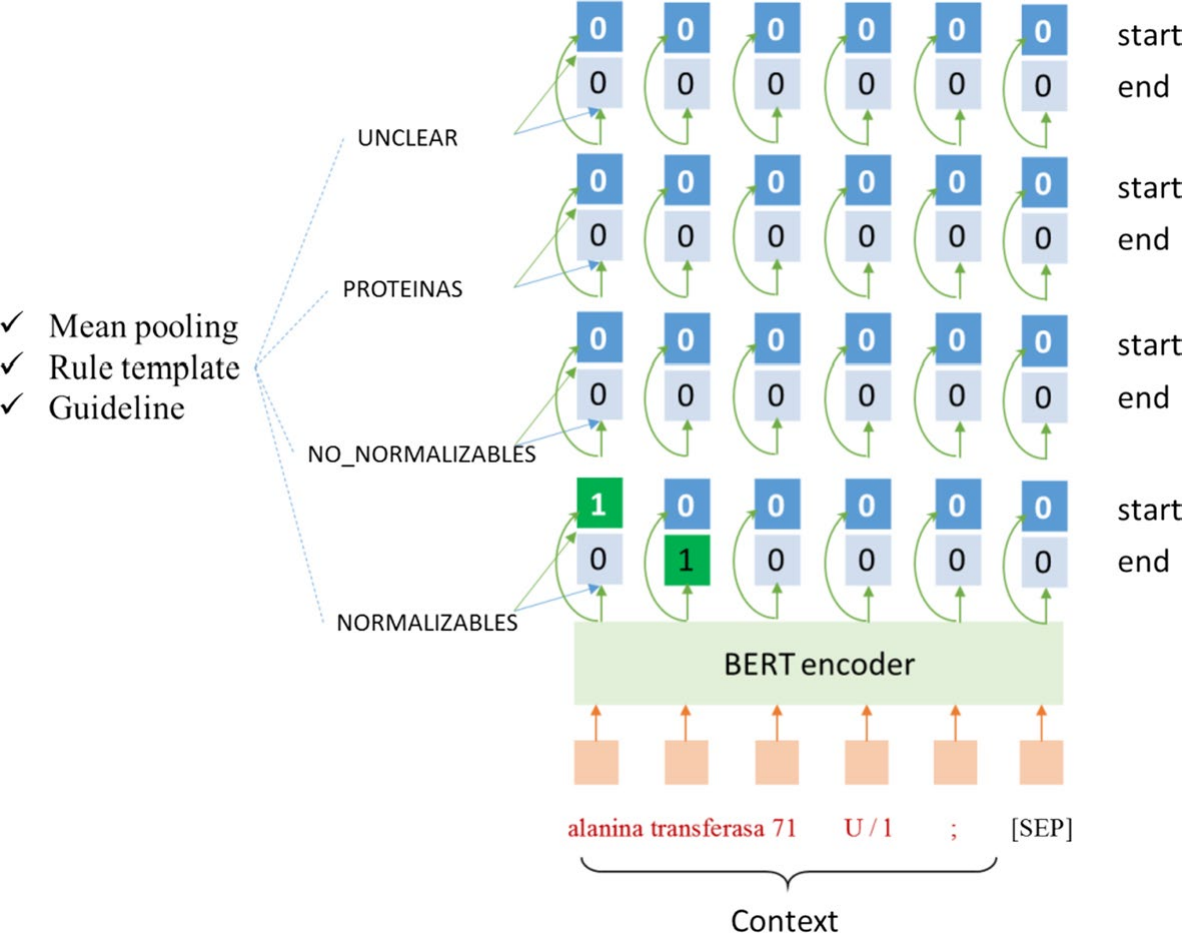
Span-level one-pass (denote as SOne) model for NER

Figure 3 shows the skeleton of the SOne model. In this model, we first use BERT to encode the input sentence *X* as ${\text{\rm Z}} \in {\mathbb{R}}^{n \times d}$ (i.e., the output of the BERT’s last layer), and then concatenate the entity definition information representation $e \in {\mathbb{R}}^{{d_{e} }}$ to all tokens, where *d*_*e*_ is the dimension of the entity definition information representation. Here, we consider three kinds of entity definition information: (1) entity mentions word embedding. each entity type definition information is represented by the mean pooling of word2vec embeddings of all tokens in all mentions of that type [42] (denoted as SOne_w2v). (2) Rule-based query. We use BERT to encode each query generated by rules (denoted as SOne_rule). (3) Annotation guideline encoded by BERT (denoted as SOne_guideline). The entity definition information enhanced sentence representation is represented as follows:

6 $$input_{SOne} = \left[ {Z, E} \right] ,$$

where $E \in {\mathbb{R}}^{{n \times d_{e} }}$ is *n* copied *e*, and [] denotes the concatenation operation.

Finally, the SOne model makes the same prediction for start position and end position as the MRC model. The only difference is that SOne has four input-shared span predictors with the same structure and different parameters, while MRC has four separate span predictors. The overall objective function of MRC and SOne is:

7 $$L = L_{start} + L_{end} ,$$

where $L_{start}$ is the start position prediction loss and $L_{end}$ is the end position prediction loss.

### Evaluation metrics

The performances of all models are measured by micro-averaged precision (P), recall (R), and F1-score (F1) under the “exact-match” criterion:

8 $$P = \frac{\# TP}{{\# \left( {TP + FP} \right)}},$$

9 $$R = \frac{\# TP}{{\# \left( {TP + FN} \right)}},$$

10 $$F1 = 2 \times \frac{P \times R}{{P + R}},$$

where TP is true positive, FP is false positive, and FN is false negative.

These measures can be calculated by the evaluation tool [43] released by the official organization of the PharmaCoNER 2019 challenge.

### Experiment setting

Following Xiong’s work [44], we first train our models on the training set and development set, and then further finetune the model for 20 epochs. The max sentence lengths of the MRC model and SOne model are set as 250 and 230, respectively. The difference in the max length is due to the query in the MRC model. The learning rate of BERT is set as 2e−5, the batch size of all models is set as 20. The dimension of entity definition information representation *d*_*e*_ is set as 300. Other parameters are set as the default. The code is available at [45].

## Results and discussion

### Performance evaluation

Table 3 presents the results of our proposed MRC and SOne model (lower part) and summarizes some reported results on the PharmaCoNER Corpus (upper part).

**Table 3:** Results on PharmaCoNER Corpus

Models	Features	Precision	Recall	F1-score
Xiong et al. [44]	Yes	0.9123	0.9088	0.9105
Stoeckel et al. [47]	No	0.9079	0.9030	0.9052
Sun et al. (2019) [48]	No	0.9046	0.8806	0.8924
Lange et al. [49]	Yes	0.8895	0.8827	0.8861
Hakala et al. [50]	No	0.8758	0.8719	0.8738
Lahuerta et al. [51]	No	0.9022	0.8366	0.8682
Sohrab et al. [52]	Yes	0.8688	0.8665	0.8676
MRC_rule		0.915	0.9055	0.9109
MRC_guideline		**0.9225**	0.9050	0.9137*
SOne (w/o entity definition)		0.9158	0.8974	0.9065
SOne_rule		0.9153	0.9088	0.912
SOne_guideline		0.9135	**0.9121**	0.9128
SOne_w2v		0.9167	0.9023	0.9094

The method with the highest F-score among all methods is highlighted in bold

* Compared with the model without any feature, this is a significant improvement (t-test < 0.05)

First, the micro-average precision, recall and F1-score of MRC_rule and MRC_guideline is 0.915, 0.9055, 0.9109 and 0.9225, 0.9050, 0.9137, respectively. Results show that both MRC_rule and MRC_guideline outperform the baseline model SOne by 0.44% and 0.72% in micro-averaged F1-score. The reason why MRC_guideline performs better than MRC_rule lies in the expertness of guideline definition. For SOne extended models, all kinds of entity definition information representation can bring improvements to the baseline model SOne. Compared with SOne, the micro-averaged F1-score of SOne_rule increases to 0.912, SOne_guideline increases to 0.9128, and SOne_w2v increases to 0.9094. The overall micro-averaged F1-score improvements of extended SOne models range from 0.29 to 0.63%.

Second, MRC-guideline outperforms all existing systems on the PharmaCoNER corpus, creating new state-of-the-art results and pushing the micro-averaged F1-score of the benchmark to 0.9137, which amounts to 0.32% absolute improvement over the top-1 system of the PharmaCoNER 2019 challenge, developed by us that using lots of features, and 1% absolute improvement over our previous system without using features [44], which is a significant improvement. We perform a significance test by comparing the model without using any feature with our MRC model or SOne model, and the results show that the improvement is significant (t-test < 0.05) [46]. This implies that entity definition information has a positive impact on entity recognition.

Third, Table 4 shows the detailed results of each entity type of MRC_guideline and SOne_guideline. Both MRC_guideline and SOne_guideline perform best on NORMALIZABLES and worst on NO_NORMALIZABLES. Though MRC_guideline outperforms SOne_guideline in terms of micro-averaged F1-score, it wrongly predicts all NO_NORMALIZABLES type. The probable reason is that queries of NORMALIZABLES and NO_NORMALIZABLES are too similar, which may confuse our models. Overall, MRC_guideline outperforms better than SOne_guideline on micro-averaged precision but worse on micro-averaged recall. Besides, we analyze all our proposed models and find that the SOne model can recognize the NO_NORMALIZABLES entities, but the MRC model cannot. It may be because that concatenation of entity definition representation benefits to few samples.

**Table 4:** Detailed results of each entity type of MRC_guideline and SOne_guideline

Entity type	Model	Precision	Recall	F1 score
NORMALIZABLES	MRC_guideline	**0.9428**	**0.9322**	**0.9375**
	SOne_guideline	0.937	**0.9322**	0.9346
NO_NORMALIZABLES	MRC_guideline	0.0	0.0	0.0
	SOne_guideline	**1.0**	**0.1**	**0.1818**
PROTEINAS	MRC_guideline	**0.8994**	0.8847	0.892
	SOne_guideline	0.8874	**0.8987**	**0.893**

The methods with the highest F-scores in each entity type are highlighted in bold

### Error analysis

Comparing with previous state-of-the-art models, our model can recognize more named entities due to the domain knowledge embedded in the entity definition information. For example, because of the introduction of the PROTEIN information, our model can recognize “timoglobulina (thymoglobulin)”, “protrombina (prothrombin)” and so on, which are ignored by previous state-of-the-art models. To visualize the effect of the added domain knowledge, we calculate the cosine similarity of some words based on their word2vec embeddings. For example, the similarity of “protrombina” and “proteínas” is more than 0.5 but has a lower similarity with “normalizar” or words in the question of the UNCLEAR type.

Though the MRC_guideline model outperforms other models, there are also some errors, mainly of the following five kinds. (1) About 20% of errors are due to the predicted entities not included in the gold test set. Although these predicted entities are the ones that have appeared, such as "vimentina (vimentin)", they are wrong because they are not officially annotated. (2) About 30% of errors are due to that the model omits some entities. (3) About 16% of the errors are because the model predicts the correct entity type, but the boundary is too long. For instance, the correct entity is "anticuerpos anticitoplasma (cytoplasmic antibodies)", but the model predicts "anticuerpos anticitoplasma de neutrófilo (antineutrophil cytoplasmic antibodies)", or the correct entity is "hormonas de crecimiento (growth hormones)", but the model predicts "hormonas de crecimiento y antidiurética (growth hormones and antidiuretics)". (4) About 20% of errors are because the model predicts the correct entity type, but the boundary is too short. For example, "tinción de auramina" is wrongly predicted as "auramina (auramine)", "anticuerpos antimembrana basal glomerular (glomerular basement membrane antibodies)" is wrongly predicted as "nticuerpos antimembrana basal (basal membrane antibodies)", and "(Ig)A-kappa" is wrongly predicted as "Ig". (5) About 10% of the errors are caused by that the model predicts the wrong entity type, and 70% of them are because that "NO_NORMALIZABLES" entity type is mistakenly predicted as "NORMALIZABLES", such as "Viekirax", "Tobradex" and "Harvoni".

## Conclusion

This paper proposed two kinds of entity definition information enhanced model, MRC and SOne for biomedical NER. Compared with the previous models, our methods do not require features and achieve state-of-the-art performance with a micro-average F1-score of 0.9137 on the PharmaCoNER Corpus. It indicates that the introduction of entity definition information is effective. In the future, we are planning to introduce more effective entity category definition information through domain knowledge graphs and to explore more valid methods to add the entity definition information, such as attention mechanism.

## Data Availability

The dataset is available on https://temu.bsc.es/pharmaconer/ [accessed on February 9, 2021].

## Abbreviations

NER: Named entity recognition
MRC: Machine reading comprehension
CRF: Conditional random fields
NLP: Natural language processing
I2B2: Informatics for Integrating Biology and the Bedside
BioCreative: Critical Assessment of Information Extraction systems in Biology
SemEval: Semantic Evaluation
CCKS: China Conference on Knowledge Graph and Semantic Computing
EHRs: Electronic health records
ME: Maximum Entropy
SSVM: Structural Support Vector Machines
HMM: Hidden Markov Models
SVM: Support Vector Machines
CNN: Convolutional Neural network
RNN: Recurrent Neural Network
LSTM: Long Short Term Memory neural network
